# Modular organization of functional brain networks in patients with degenerative cervical myelopathy

**DOI:** 10.1038/s41598-024-58764-7

**Published:** 2024-04-13

**Authors:** Ziwei Shao, Yongming Tan, Yaru Zhan, Laichang He

**Affiliations:** 1 Department of Radiology, The First Affiliated Hospital of Nanchang University, Nanchang, 330006 China; 2 Clinical Research Center for Medical Imaging In Jiangxi Province, Nanchang, China

**Keywords:** Cervical spondylotic myelopathy, Graph theory analysis, Modularity analysis, Network interactions, Resting fMRI, Neurological disorders, Predictive markers

## Abstract

Previous studies have indicated that brain functional plasticity and reorganization in patients with degenerative cervical myelopathy (DCM). However, the effects of cervical cord compression on the functional integration and separation between and/or within modules remain unclear. This study aimed to address these questions using graph theory. Functional MRI was conducted on 46 DCM patients and 35 healthy controls (HCs). The intra- and inter-modular connectivity properties of the whole-brain functional network and nodal topological properties were then calculated using theoretical graph analysis. The difference in categorical variables between groups was compared using a chi-squared test, while that between continuous variables was evaluated using a two-sample t-test. Correlation analysis was conducted between modular connectivity properties and clinical parameters. Modules interaction analyses showed that the DCM group had significantly greater inter-module connections than the HCs group (DMN-FPN: t = 2.38, *p* = 0.02); inversely, the DCM group had significantly lower intra-module connections than the HCs group (SMN: t = − 2.13, *p* = 0.036). Compared to HCs, DCM patients exhibited higher nodal topological properties in the default-mode network and frontal–parietal network. In contrast, DCM patients exhibited lower nodal topological properties in the sensorimotor network. The Japanese Orthopedic Association (JOA) score was positively correlated with inter-module connections (r = 0.330, FDR *p* = 0.029) but not correlated with intra-module connections. This study reported alterations in modular connections and nodal centralities in DCM patients. Decreased nodal topological properties and intra-modular connection in the sensory-motor regions may indicate sensory-motor dysfunction. Additionally, increased nodal topological properties and inter-modular connection in the default mode network and frontal-parietal network may serve as a compensatory mechanism for sensory-motor dysfunction in DCM patients. This could provide an implicative neural basis to better understand alterations in brain networks and the patterns of changes in brain plasticity in DCM patients.

## Introduction

Degenerative cervical myelopathy (DCM) is the leading cause of spinal cord dysfunction in adults worldwide. DCM encompasses various acquired (age-related) and congenital pathologies related to degeneration of the cervical spinal column, including hypertrophy and/or calcification of the ligaments, intervertebral discs and osseous tissues, these pathologies narrow the spinal canal, leading to chronic spinal cord compression and disability^1,2^. Injuries caused by spinal cord compression include reversible and irreversible injuries, with the majority of patients demonstrating recovery of neurologic function postoperatively, whereas irreversible injuries caused by longer-term compression have unsatisfactory postoperative outcomes^3^.

The plasticity of the mammalian sensorimotor system is the basis of motor learning ability and the ability to relearn after injury; corticomotor and somatosensory sensations show spontaneous reorganization after spinal cord injury; this plasticity contributes to the recovery of motor and sensory functions and provides targets for therapeutic interventions^4^. At the core of sensorimotor integration after injury is the strengthening of circuits weakened by injury or the use of complementary pathways to compensate for the function of circuits lost through injury^5^. Previous research has reported local measurement of spontaneous neural activity changes linked to DCM^6,7^. Our group also performed seed-based functional connectivity analysis using the thalamus as the seed region to obtain more detailed information about the functional connectivity changes between the thalamus and the cortex in DCM^8,9^. However, an increasing number of studies demonstrate that the brain accomplishes behavioral change complex hierarchy of functional interactions between several human regions^10^. Spinal cord compression altered the whole-brain network organization in patients with DCM remains an open question. To further clarify this issue, further research should focus on the modular organization of brain networks.

As in many complex systems, brain networks demonstrate modularity and modules are often made up of anatomically neighboring and/or functionally related cortical regions, each of which comprises a number of nodes that are densely intra-connected to each other but sparsely inter-connected to nodes in other modules^11,12^. The inter-modular connectivity enables functional segregation promoting, as each module serves a specific function. And the intra-modular connectivity allows functional integration between modules through interaction^13^. Modularity analysis can reveal the intra- and inter-modular connectivity properties of the brain and reflect the integration and segregation of brain network^14^.

Graph theory methods can offer important new insights into the structure and function of networked brain systems, which is readily represented as a graph of nodes and edges^15^. We further examined the nodal topological property measuring the proportion of inter- and intra-modular connections^16^. Thus, the method is used to conduct a more complete insight into brain plasticity by assessing interactions in full-scale brain network, rather than specific activity in any one functional area.

Based on this, the novelty of this study aims to explore the changes of modular connections of the whole-brain functional network, opening up new avenues to better understanding brain plasticity in DCM patients. We hypothesized that some brain network modules, such as the sensorimotor network, would become less segregated, mediating sensorimotor dysfunction, and that between-module connectivity would increase in other networks to compensate for the dysfunction.

## Methods

### Participants

This study was performed following the Declaration of Helsinki. The study was approved by the Ethics Committee of the First Affiliated Hospital of Nanchang University (2014–037), and written informed consent was obtained from each participant before the study. All experiments were performed in accordance with relevant guidelines and regulations.

A total of 48 DCM patients and 37 healthy controls (HCs) were initially selected for this study. Data from two patients and two controls were excluded based on head motion greater than 3 mm translation or 3 degrees in any direction during the fMRI scan. A total of 81 participants were finally recruited between May 2014 and May 2019, comprising of 46 DCM patients (29 males and 17 females; mean age 49.61 ± 6.65 years; range 36 to 62 years) and 35 HCs of level-matched age, sex, and education matched (24 males and 11 females; mean age 50.03 ± 9.40 years; range 30–68 years).Inclusion criteria were: (1) were aged between 22 and 65 years; (2) volunteered to enroll in the study; (3) an indication for cord compression on a cervical spine MRI, such as cervical spondylosis, herniated discs or ossification of the posterior longitudinal ligament; and (4) existence of myelopathy symptoms. Exclusion criteria were: (1) refusal to enroll; (2) traumatic cord compression; and (3) a history of neurological disorders such as cerebrovascular disease or tumor. . All patients should complete Japanese Orthopaedic Association (JOA) Scores and Neck Disability Index (NDI) assessment.

### MRI data acquisition

All participants performed 3.0 T MRI (Siemens Trio Tim, Erlangen, Germany) scan with a 4-channel cervical coil and an 8-channel head coil. Before the scan, subjects were asked to stay awake without intense mental activity, close their eyes, and lie comfortably on the examination bed. Sagittal and axial images of the brain and cervical spinal cord were collected, including conventional T1WI, T2WI, and fluid attenuated inversion recovery T2WI. Conventional MR scan was performed to diagnose and exclude brain disorders (such as tumor, cerebral infarction, hemorrhage, encephalomalacia foci) and cervical spinal cord disease (such as multiple sclerosis, amyotrophic lateral sclerosis, and intramedullary tumors). (1) High-resolution anatomic images of brain were acquired by 3D T1weighted spoiled gradient recall sequence with the following parameters: repetition time (TR) = 1900 ms, echo time (TE) = 2.26 ms, flip angle = 9°, field of view (FOV) = 256 × 256 mm, matrix = 256 × 256, slice thickness = 1 mm, number of slices = 176, voxel size = 1.0 × 1.0 × 1.0 mm^3^, and interslice gap = 0.5 mm. (2) Gradient-recalled echo-planar imaging (GRE-EPI) sequence parameters of brain were as followed: TR/TE = 2000 ms/30 ms, flip angle = 90°, FOV = 200 × 200 mm, matrix = 64 × 64, number of slices = 30, slice thickness = 4 mm, interslice gap = 1.2 mm, voxel size = 3.0 × 3.0 × 4.0 mm3, and 240 time points (8 min 6 s). (3) C1–C7 cervical spinal cord DTI parameters were acquired by single-shot spin echo echo-planar image (SS-SE-EPI): TR = 5000 ms, TE = 111 ms, FOV = 109 × 109 mm, number of excitations (NEX) = 2, matrix = 128 × 124, slice thickness = 7 mm, voxel size = 0:7 × 0:7 × 7 mm, and diffusion encoding occurred in 20 noncollinear and noncoplanar diffusion directions, with b = 600 s/mm^2^.

### Data preprocessing

The R-fMRI data preprocessing was performed using the GRETNA toolbox (http://www.nitrc.org/projects/gretna/) based on SPM12 (http://www.fil.ion.ucl.ac.uk/spm/software/spm12/). The preprocessing procedure included (1) discarding the first 10 time points of the images for MR signal equilibrium, (2) slice timing correction, (3) head motion correction, (4) space normalization, registering functional data to the corresponding structural T1-weighted image and aligning the T1 images to Montreal Neurological Institute (MNI) space with resampling to a voxel size of 3 × 3 × 3 mm^3^, and (5) nuisance covariate regressions were performed (including 24 motion parameters, white matter, and cerebrospinal fluid signals. (6) The resulting images were further temporally band-pass filtered (0.01–0.1 Hz) to reduce the effects of low-frequency drift and high-frequency physiological noise. Two patients and two controls were excluded based on the criterion of a displacement > 3 mm or an angular rotation > 3 degrees in any direction.

### Network construction

To obtain defined nodes, we applied a functional template as proposed in a previous study^17^. This functional template can parcellate the brain into 160 functionally segregated regions of interest (ROIs) that cover most of the cerebral cortex and cerebellum. The set of ROIs (3 mm diameter spheres) were generated using the peak coordinates derived from a series of meta-analyses of fMRI activation studies^17^. We chose this functional template for defining the functional network nodes given that it has been broadly applied to examine whole-brain functional connectivity during resting-state^18^. Average time courses from each ROI were extracted and pair-wise Pearson correlation coefficients were computed between these ROIs. There are no available gold-standard criteria to determine a precise sparsity threshold. Therefore, we explored graph correlation matrices with a wide range of sparsity thresholds from 0.04 to 0.4, with an interval of 0.01. This resulted in 37 sparse connectivity matrices. The minimum and maximum values of the sparsity threshold were established to ensure that threshold networks were estimable for small-worldness (σ) scalar and that σ was greater than 1.1.

### Graph theory analysis

Graph analysis was conducted using functions implemented in the GRETNA toolbox (http://www.nitrc.org/projects/gretna/) based on SPM12 (http://www.fil.ion.ucl.ac.uk/spm/software/spm12/), and the results of node-assignments were presented by BrainNet Viewer (http://www.nitrc.org/projects/bnv/).

**Figure 1:**
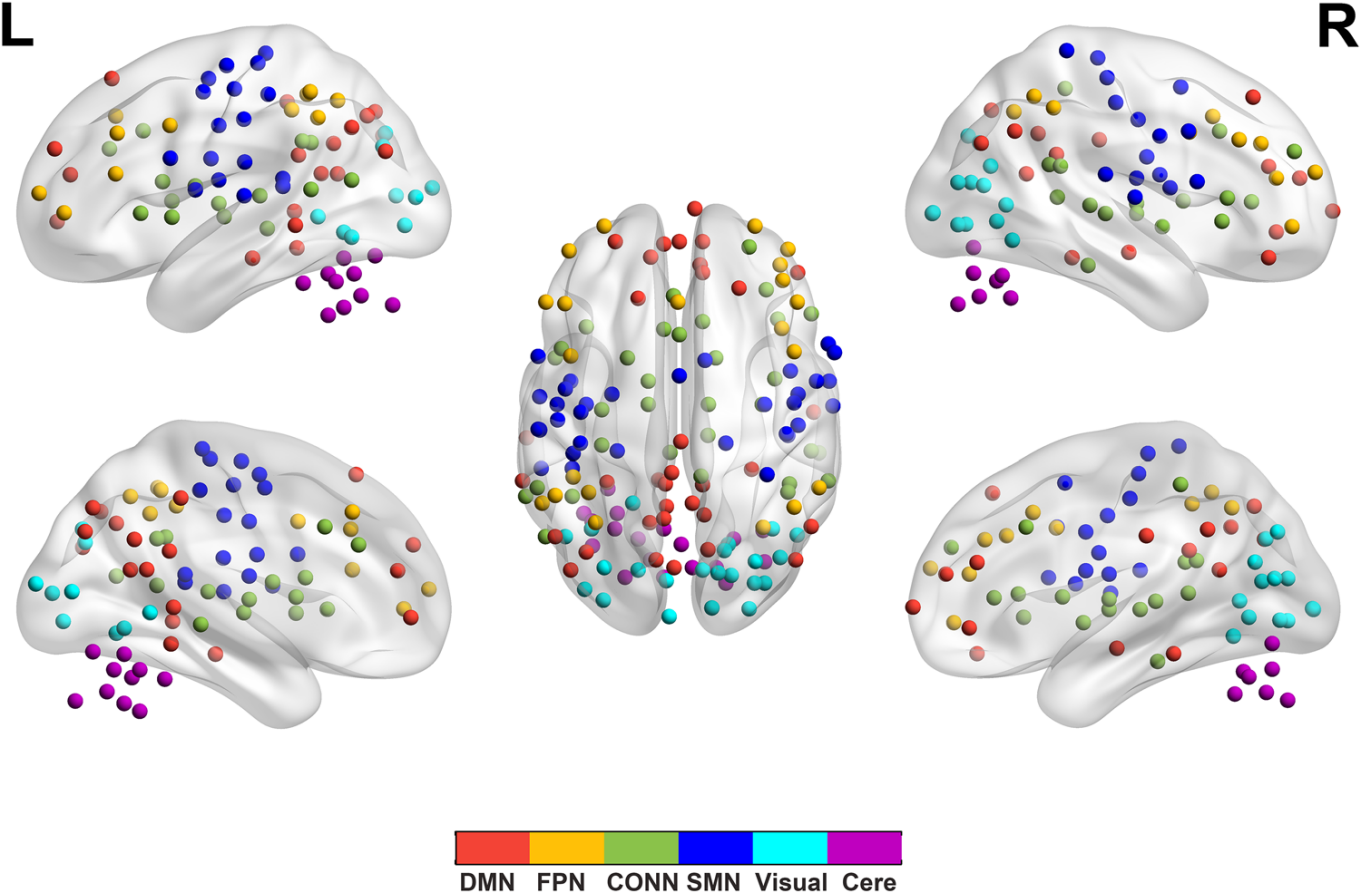
Module partitions. DMN, the default-mode network; FPN, the frontal–parietal network; CONN, the cingulo-opercular network; SMN, the sensorimotor network; Visual, the visual network; Cere, the cerebellum network; L, left; R, right.

According to the study of Dosenbach et al.^17^, the 160 ROIs have been assigned into six functional modules, corresponding to the default-mode network (DMN), frontal–parietal network (FPN), cingulo-opercular network (CONN), sensorimotor network (SMN), visual network, and the cerebellum (Fig. 1). After module decomposition, to find modules that were representative of both groups, we extracted nodes that belonged to the same module in both groups as representative of that module for both groups. Next, we computed the inter-/intra-module connections for each module and each subject. We analyzed the six modules interaction area under the curve (AUC) over the whole range of the sparsity threshold. We calculated the nodal topological properties included degree centrality (Dc), nodal efficiency (Ne), betweenness centrality (Bc) and participant coefficient (PC) of all nodes that were part of exceptionally connected modules. These network properties have previously been defined by Zhang et al.^19^. Moreover, the area under the curve (AUC) of all network metrics was constructed over the whole range of the sparsity threshold. The AUC provides a summarized scalar for the topological characterization of brain networks, independent of a single threshold selection, sensitive in detecting topological alterations of brain disorders, and unravel between-group differences in network organization.

### Statistical analysis

#### Differences in demographic and clinical variables

Statistical analyses were conducted using SPSS 25.0 (IBM). The difference in categorical variables between groups was tested and compared using a chi-squared test, while that between continuous variables was evaluated using nonparametric permutation tests.

#### Group comparisons based on modular connections and nodal topological metrics

To evaluate differences of intra- and inter-modular connectivity properties and nodal topological properties between DCM and HCs groups, we used a two-sample t-test (Bonferroni *p* value < 0.05).If between group significant differences were observed in any modules, then partial correlation analysis was conducted to assess the relationships between modularity, the Japanese Orthopedic Association (JOA) score, and Neck Disability Index (NDI) score in the DCM group with age and gender as covariates The significance levels were set at *p* < 0.05 (FDR corrected).

## Results

### Demographics and clinical characteristics

There was no significant difference in sex (*p* = 0.610) and age (*p* = 0.782) between DCM patients and HCs. DCM patients had a mean symptom duration of 8.72 ± 4.54 months and mean JOA score of 11.22 ± 2.36 (Table 1**)**.

**Table 1:** Demographic data and clinical measures for the degenerative cervical myelopathy patients and healthy controls.

	DCM	HCs	*p*-value
Age (year)	49.61 ± 6.65	50.03 ± 9.40	0.782
Sex (M/F)	29/17	24/11	0.610
Duration (month)	8.72 ± 4.54	NA	-
NDI	0.32 ± 0.11	NA	–
JOA	11.22 ± 2.36	NA	–
Motor Upper	2.24 ± 0.70	NA	-
Motor Lower	2.57 ± 1.13	NA	-
Sensory function	3.59 ± 1.20	NA	-
Trunk sensation	2.91 ± 0.29	NA	-

*DCM* degenerative cervical myelopathy, *HCs* healthy controls, *NDI* Neck Disability Index, *JOA* Japanese Orthopedic Association.

### Changes in characteristics over module

**Figure 2:**
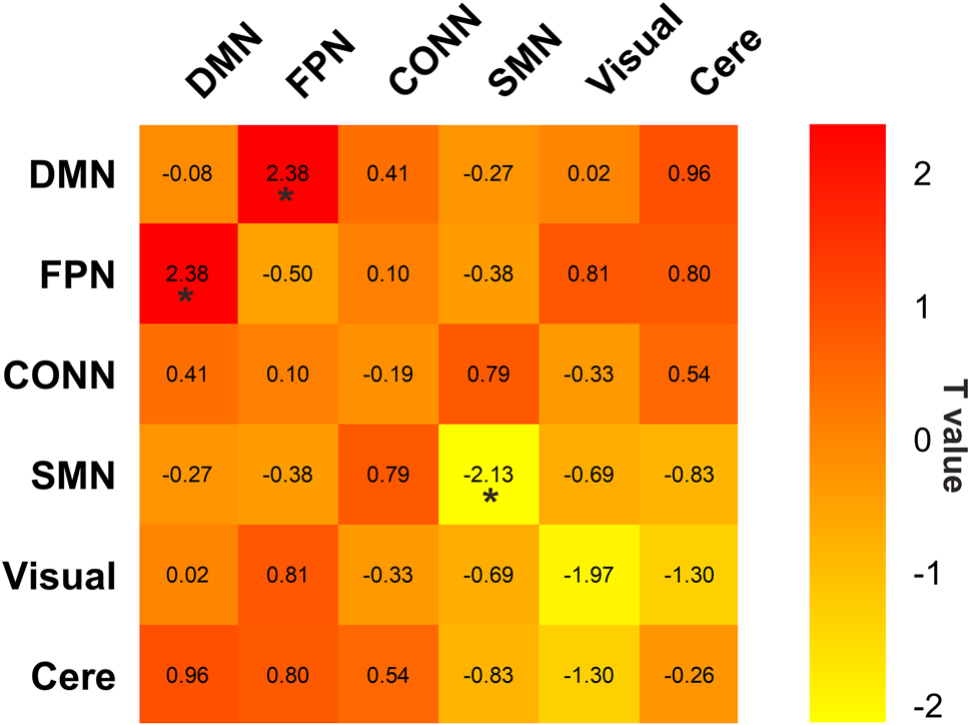
Relative to the HCs group, the DCM patients showed significantly lower intra-module connections within SMN and greater inter-module connections between DMN and FPN. *p-corrected < 0.05.

Modules interaction analyses showed that the DCM group had significantly greater inter-module connections than the HCs group (DMN-FPN: t = 2.38, *p* = 0.020); On the contrary, the DCM group had significantly less intra-module connections than the HCs group (SMN: t = − 2.13, *p* = 0.036) (Fig. 2).

### Changes in regional nodal characteristics

**Figure 3:**
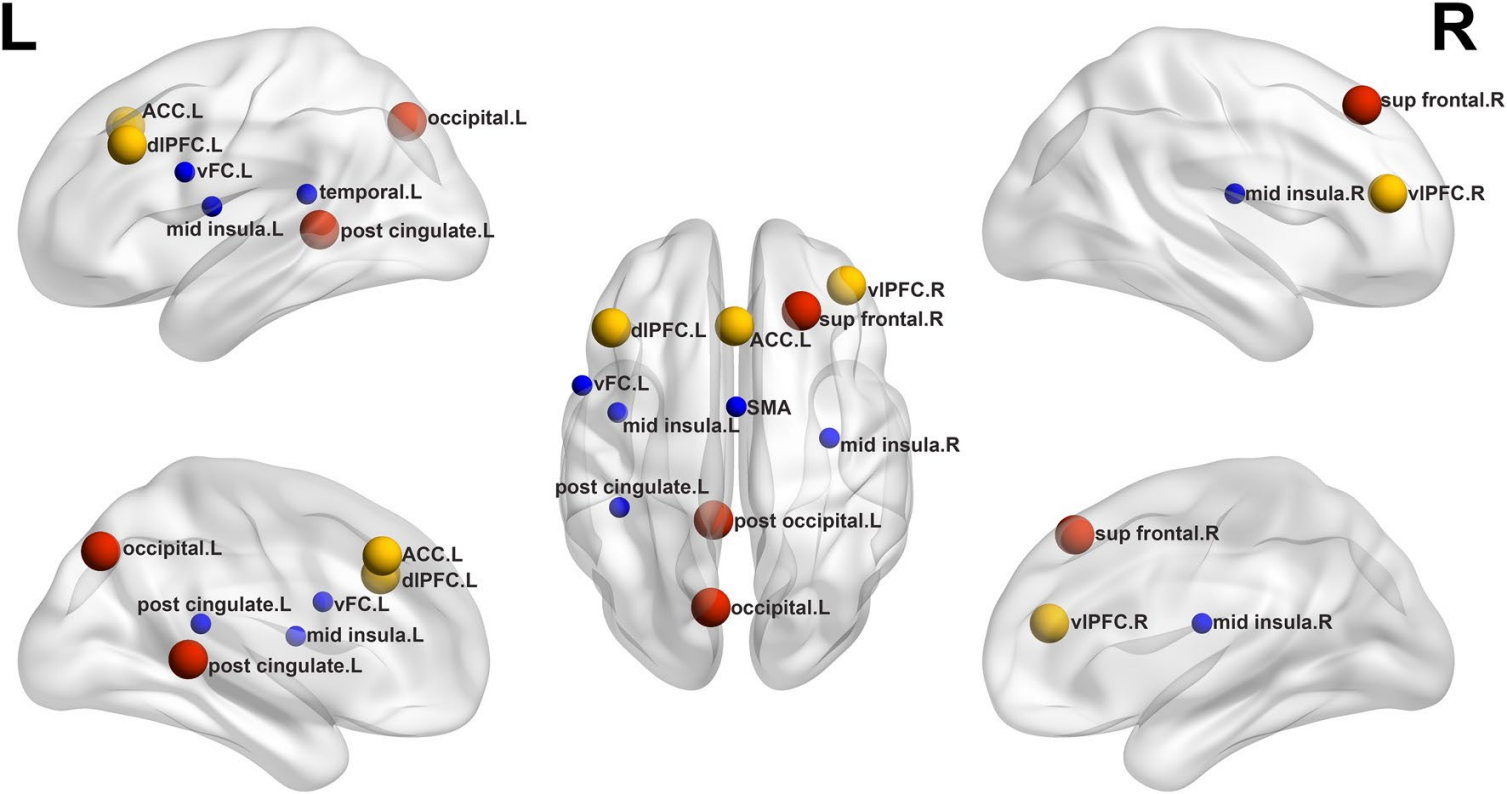
Between-group comparisons of nodal topological properties of all nodes that were part of exceptionally connected modules. Red circles represent DMN, yellow circles represent PFN, blue circles represent SMN. Bigger and smaller circles represent higher and lower nodal topological properties, respectively, as observed in DCM patients compared with healthy controls. superior frontal gyrus (sup frontal); ventrolateral prefrontal cortex (vlPFC); anterior cingulate cortex (ACC); dorsolateral prefrontal cortex (dlPFC); ventral frontal cortex (vFC); supplementary motor cortex (SMA); R, right; L, left.

Compared to HCs, DCM patients exhibited higher nodal topological properties in the right superior frontal gyrus, left post cingulate gyrus, left occipital, right ventrolateral prefrontal cortex (vlPFC), left anterior cingulate cortex (ACC) and left dorsolateral prefrontal cortex (dlPFC). In contrast, compared to the HCs group, DCM patients exhibited lower nodal topological properties in the ventral frontal cortex (vFC), SMA, bilateral mid insula and left temporal (Table 2 and Fig. 3).

**Table 2:** Regions showing abnormal nodal topological properties in DCM patients as compared with healthy controls.

Brain regions	*p* values			
	Bc	Dc	Ne	PC
DCM > HCs				
Right sup frontal	**0.026**	0.922	0.863	0.837
Left post cingulate	0.090	**0.005**	**0.005**	**0.042**
Left occipital	0.073	**0.017**	0.055	0.359
Right vlPFC	0.607	0.579	0.417	**0.004**
Left ACC	0.079	0.095	0.161	**0.002**
Left dlPFC	0.593	0.613	0.654	**0.014**
DCM < HCs				
Left vFC	0.361	0.102	**0.032**	0.112
SMA	0.092	0.538	0.470	**0.010**
Left mid insula	**0.006**	0.054	0.059	0.054
Right mid insula	**0.041**	0.481	0.377	0.781
Right temporal	0.970	0.080	0.048	**0.016**

Regions were considered abnormal in DCM patients if they exhibited significant between-group differences (Bonferroni *p* < 0.05). DCM degenerative cervical myelopathy, HCs healthy controls. superior frontal gyrus (sup frontal); ventrolateral prefrontal cortex (vlPFC); anterior cingulate cortex (ACC); dorsolateral prefrontal cortex (dlPFC); ventral frontal cortex (vFC); supplementary motor cortex (SMA).

Significant values are in bold.

### Relationships between modular measures and clinical variables

**Figure 4:**
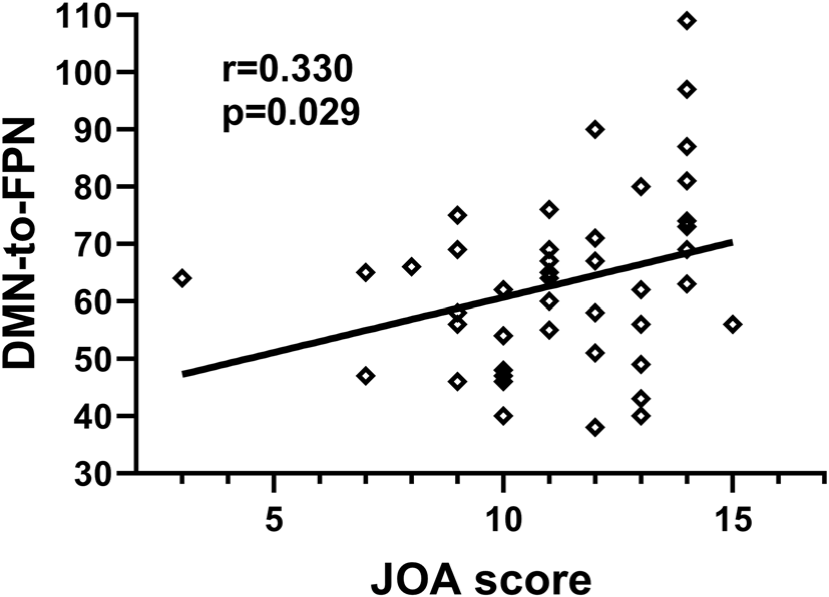
Correlation analysis of modular connection and clinical variables in DCM patients. The JOA score positively correlated with inter-module connections between DMN and FPN.

The Japanese Orthopedic Association (JOA) score was positively correlated with inter-module connections (r = 0.330, FDR *p* = 0.029) but not correlated with intra-module connections. and there was also no correlation between module connections and NDI score (Fig. 4).

## Discussion

In this current study, modules interaction analyses showed that, in the DCM group, intra-module connections decreased significantly within the SMN, but there was no correlation with the JOA score. In contrast, inter-module connections between the DMN and the frontal–parietal network increased significantly, which was positively correlated with the JOA score. Then we calculated the nodal topological properties of all nodes that were part of exceptionally connected modules. The nodal topological properties were higher in DCM patients than in controls in the right superior frontal gyrus, left post cingulate cortex, left occipital, right ventrolateral prefrontal cortex, left anterior cingulate cortex and left dorsolateral prefrontal cortex. DCM patients had lower nodal topological properties than in controls in the sensorimotor network.

One finding of this study is that intra-module connections and nodal topological properties were lower within the SMN network in the DCM group. Previous studies demonstrated alterations in activation volume^20,21^ and functional connectivity^22^ in the sensorimotor cortex (SMC), an important brain network in patients with DCM. Advanced study also reported metabolite^23^ and cerebral blood flow^24^ changes in both the motor and sensory cortices. We have found that the modularity within the SMN is reduced in DCM patients, indicating that functional brain networks become less specific. Functionally, this pattern in DCM patients may be related to a drop in sensorimotor function. Following the reduced modularity, the local efficiency within the SMN network also decreased, which were mainly located in the were mainly located in the vFC, SMA, bilateral mid insula and left temporal. We report for the first time changes in the ventral frontal cortex, but we still don't know its role in DCM. There is increasing evidence indicating that SMA is crucial for gait initiation prior to voluntary movement^25^ and Ryan et al.^26^ also indicated that the volume of activation of the SMA decreased in DCM patients. Galhardoni et al.^27^ have found that insula neurons can modulate different dimensions of pain. A review suggests that temporal lobe linked to memory, emotion and executive functions^28^. We speculate that patient motor dysfunction may be related to the decline of its executive capacity. In summary, changes in the sensorimotor network may underlie the production of clinical symptoms.

On the contrary, topological properties of nodes within the DMN and FPN, as well as inter-network connections between the DMN and FPN, were increased in the DCM group compared to the healthy control group. The significance of the DMN is role in cognition and it's been linked to a variety of mental illnesses^29^. Further, recent studies have intermittently identified symptoms beyond the cord, including depression, anxiety and cognitive deficits^30,31^. The DMN also provides a spatial framework for multiple large-scale networks^32^. Our results also showed increased nodal topological properties in the FPN including right vlPFC, left ACC and left dlPFC. The lateral prefrontal cortex(LPFC) showed significant capability in coding of visual information, behavioral decision and widespread information exchange ^33–35^, reflecting multiple aspects and levels^36–38^ of executive control. And the ACC is a key region for pain processing and can modulate the neuronal activity for neuropathic pain^39^. Increased node strength indicates enhanced physiological functioning, suggesting a compensatory role for sensory-motor impairment in DCM.

Additionally, we found higher JOA score was associated with greater inter-modular connection. It indicates that milder clinical symptom of DCM may be due to compensatory effects of brain reorganization processes. The increased inter-module connections represented strengthening connections between the DMN and FPN and information transmission. Specifically, the compression of the cervical cord led to decreased modularity and increased modular integrity in cerebrum.

Several limitations in the current study are noteworthy. First, changes in patients after decompression surgery were not evaluated and longitudinal studies could be conducted in the future to better establish a cause and effect relationship between the clinical/imaging variables analyzed and surgical outcome. Additionally, we found the connection of the default-mode network was altered in DCM patients compared to controls. As this network plays an important role in cognition, more scales assessing cognitive function should have been included in our analysis. Finally, our study did not classify the severity of spinal cord compression, which may be associated with brain plasticity.

In conclusion, decreased nodal topological properties and intra-modular connection in the sensory-motor regions may represent the potential physiological basis for sensory-motor impairment. Furthermore, increased nodal topological properties and inter-modular connection in the DMN and the FPN of DCM patients could be a compensatory for sensory-motor dysfunction in DCM. This could provide an implicative neural basis to better understand alterations in brain networks and the patterns of changes in brain plasticity in DCM patients.

## Data Availability

The datasets generated during this study are available from the corresponding author upon reasonable request.
